# Prevalence of molecular markers of anti-malarial drug resistance in *Plasmodium vivax *and *Plasmodium falciparum *in two districts of Nepal

**DOI:** 10.1186/1475-2875-10-75

**Published:** 2011-04-01

**Authors:** Samir Ranjitkar, Mette L Schousboe, Thomas Thyge Thomsen, Madhav Adhikari, Christian MO Kapel, Ib C Bygbjerg, Michael Alifrangis

**Affiliations:** 1 Centre for Medical Parasitology, Department of International Health, Immunology and Microbiology, Faculty of Health Sciences, University of Copenhagen, Copenhagen, Denmark; 2 Central Department of Microbiology, Tribhuvan University, Kathmandu, Nepal; 3 Department of Agriculture and Ecology, Faculty of Life Sciences, University of Copenhagen, Copenhagen, Denmark

## Abstract

**Background:**

Sulphadoxine-pyrimethamine (SP) and chloroquine (CQ) have been used in treatment of falciparum and vivax malaria in Nepal. Recently, resistance to both drugs have necessitated a change towards artemisinin combination therapy (ACT) against *Plasmodium falciparum *in highly endemic areas. However, SP is still used against *P. falciparum *infections in low endemic areas while CQ is used in suspected cases in areas with lack of diagnostic facilities. This study examines the prevalence of molecular markers of CQ and SP resistance in *P. falciparum *and *Plasmodium vivax *to determine if high levels of *in vivo *resistance are reflected at molecular level as well.

**Methods:**

Finger prick blood samples (n = 189) were collected from malaria positive patients from two high endemic districts and analysed for single nucleotide polymorphisms (SNPs) in the resistance related genes of *P. falciparum *and *P. vivax *for CQ (*Pfcrt, Pfmdr1, Pvmdr1*) and SP (*Pfdhfr, Pfdhps, Pvdhfr*), using various PCR-based methods.

**Results and discussion:**

Positive *P. vivax *and *P. falciparum *infections were identified by PCR in 92 and 41 samples respectively. However, some of these were negative in subsequent PCRs. Based on a few *P. falciparum *samples, the molecular level of CQ resistance in *P. falciparum *was high since nearly all parasites had the *Pfcrt *mutant haplotypes CVIET (55%) or SVMNT (42%), though frequency of the *Pfmdr1 *wild type haplotype was relatively low (35%). Molecular level of SP resistance in *P. falciparum *was found to be high. The most prevalent *Pfdhfr *haplotype was double mutant CNRNI (91%), while frequency of *Pfdhps *double mutant SGEAA and AGEAA were 38% and 33% respectively. Combined, the frequency of quadruple mutations (CNRNI-SGEAA/AGEAA) was 63%. Based on *P. vivax *samples, low CQ and SP resistance were most likely due to low prevalence of *Pvmdr1 *Y976F mutation (5%) and absence of triple/quadruple mutations in *Pvdhfr*.

**Conclusions:**

Based on the limited number of samples, prevalence of CQ and SP resistance at molecular levels in the population in the study area were determined as high in *P. falciparum *and low in *P. vivax*. Therefore, CQ could still be used in the treatment of *P. vivax *infections, but this remains to be tested *in vivo *while the change to ACT for *P. falciparum *seems justified.

## Background

Malaria is one of the major health problems in South-East Asia where 1.3 billion people (76% of total population) are at risk, causing around 120,000 deaths yearly [1]. *Plasmodium vivax *malaria is the most widespread and prevalent in this region but large knowledge gaps still exits that preventing the assessment of the technical feasibility of its elimination still exist [2]. In Nepal, roughly 80% (22.5 million) of the population lives in malaria endemic areas of which seven million reside in highly endemic areas [3]. *P. vivax *is the predominant species that causes around 80-90% of the total malaria cases while *Plasmodium falciparum *is the main cause of malaria epidemics in Nepal [4]. Malaria transmission may occur throughout the year but mostly from March to November with peaks in June, July and August [5]. Chloroquine (CQ) is the first-line drug for the treatment of *P. vivax *infections as well as for the treatment of suspected *P. falciparum *infections in situations where diagnosis is unavailable; sulphadoxine-pyrimethamine (SP) has been the drug of choice for laboratory confirmed uncomplicated *P. falciparum *infections. Recently, artemisinin combination therapy (ACT) was introduced in 13 high endemic areas, replacing SP, as treatment for laboratory confirmed uncomplicated *P. falciparum *due to reports of high SP resistance [4].

Anti-malarial drugs have been one of the most important tools in the control of malaria over the last 50 years [6,7]. However, a major impediment to this strategy is the capacity of the parasites to develop drug resistance. This resistance has spread almost globally with regard to CQ and SP [7,8]. In Nepal, CQ resistance to *P. falciparum *was first observed in 1984, then in 1986, 1987 and 1988 [9]. CQ resistance in *P. vivax *has not been recorded. SP resistance in *P. falciparum *was first recorded in 1996 and then again in 2003 [9,10]. These studies were based on reports of treatment failures. Molecular markers of drug resistance, which are now commonly applied as an adjunct in the surveillance of anti-malarial drug resistance, have had only been used limited in Nepal. The advantage of using molecular markers of drug resistance is that the level of drug resistance can be studied retrospectively, such as after a drug has been abandoned due to reports of high levels of drug resistance *in vivo*.

CQ resistance is associated with single nucleotide polymorphisms (SNPs) in the *P. vivax *multidrug resistance gene 1 (*Pvmdr1*) at codon (c) 976 (Y976F) [11] and the *P. falciparum *chloroquine resistance transporter gene (*Pfcrt*) at c76 (K76T)[12,13]. In *P. falciparum*, certain mutant haplotypes at c72-76 are highly prevalent globally, namely CVIET and SVMNT [14]. Additionally, SNPs at c86, c184 and c1246 in *P. falciparum mdr-1 *(*Pfmdr1*) have been associated with CQ resistance [6,15]. However, its role is considered as modulatory when SNPs in *Pfmdr1 *occur with the *Pfcrt *K76T mutation [7].

Resistance to SP is conferred by SNPs in the genes coding for dihydrofolate reductase (*dhfr*) and dihydropteroate synthetase (*dhps*) in both *P. vivax *[16,17] and *P. falciparum *[18,19]. Although SP is not used deliberately against *P. vivax *infections, exposure can occur because mixed infections are common and often misdiagnosed in Asia [20-22]. In *P. vivax*, SNPs at c57 (F57L), c58 (S58R), c61 (T61M) and c117 (S117N) in *Pvdhfr *are associated with pyrimethamine (PYR) resistance [23,24]. For *P. falciparum*, SNPs at c51 (N51I), c59 (C59R), c108 (S108N) and c164 (I164L) of *Pfdhfr *are associated with PYR resistance [7,25] while sulphadoxine resistance is associated with SNPs at c436 (S436A), c437 (A437G), c540 (K540E), c581 (A581G) and c613 (A613S/T) [8,25].

In Nepal, a limited study of molecular markers of anti-malarial drug resistance only in *P. falciparum *has been carried out [26]. Therefore, in light of the changing drug policy in Nepal towards using ACT; artemether-lumefantrine (AL) in some areas for *P. falciparum *and to fill some of the gaps in the knowledge of resistance to commonly used anti-malarials, the present study investigated the prevalence of molecular markers of anti-malarial drug resistance in both species by analysing the SNPs of *Pfcrt, Pfmdr1, Pfdhfr *and *Pfdhps *in *P. falciparum *and *Pvmdr1 *and *Pvdhfr *in *P. vivax *using samples from patients living in two highly endemic districts of Nepal.

## Methods

### Sample collection

**Figure 1:**
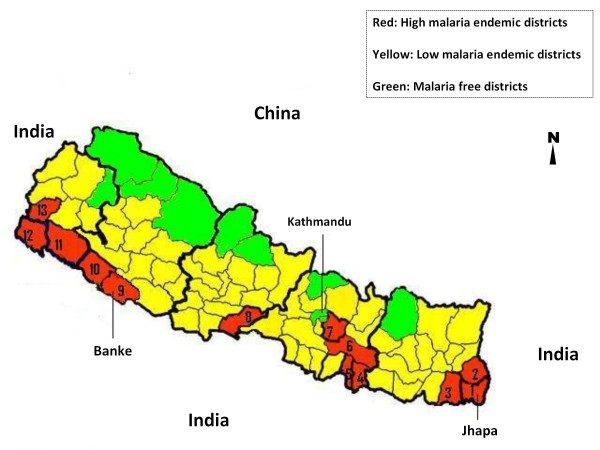
**Distribution of malaria in Nepal**. Distribution of malaria in 75 districts of Nepal. Jhapa (1) and Banke (9) districts are the study sites. High and low malaria endemic districts are categorized on the basis of abundance of two principle vectors; *Anopheles minimus *and *Anopheles fluviatilis*, and malaria transmission rate: where annual malaria transmission rate are ≥ 1/1,000 and 0- 1/1,000 population respectively [5,53]. The figure has been modified from [54] to provide more detail.

Ethical clearance was obtained from the Nepal Health Research Council, Kathmandu, for the study (Ref. No. 266). After obtaining informed consent from either the patient or legal guardian, blood samples were collected from patients attending private or government hospitals or health posts in Jhapa (n = 146) and Banke (n = 43) districts in Eastern and Western Nepal, respectively (Figure 1). Malaria is highly endemic in both districts and are two of the 13 districts where ACT (AL) was implemented in 2004 [4]. These districts share borders with the Indian states of Uttar Pradesh, Bihar and Assam where malaria is endemic [5]. Finger prick blood samples were collected either on Whatman no. 3 filter paper or as blood smear slides from malaria positive patients confirmed by microscopy or rapid diagnostic test.

### Genetic characterization of the parasites

Extraction of DNA from filter paper and blood smear slides were done by chelex method as described in [27] and [28] respectively. *Plasmodium *species (either *vivax *or *falciparum*) were differentiated by nested polymerase chain reaction (PCR) as in [29] and identification of *Plasmodium malariae *and *Plasmodium ovale *were done as previously [30]. Analysis of SNPs at *Pvmdr1 *was done by sequencing the nested PCR products as in [31]. Analysis of SNPs at *Pvdhfr *was performed by sequence specific oligonucleotide probes- enzyme-linked immunosorbent assay (SSOP-ELISA) as in [32] and for *Pfcrt, Pfdhfr *and *Pfdhps*, analysis were done by SSOP-ELISA as previously described in [33]. Finally, *Pfmdr1 *was analysed by PCR-restriction fragment length polymorphism (RFLP) as described elsewhere [34,35].

## Results

Of the 189 samples, PCR identified 113 samples (60%) positive for *Plasmodium*, of which 72 (64%) and 21 (19%) samples were *P. vivax *and *P. falciparum *respectively while 20 (17%) of the samples carried mixed *P. falciparum/P. vivax *infections (Table 1). No *P. malariae *or *P. ovale *infections were detected. Positive samples from Banke were few (11%, n = 12) when compared to Jhapa (89%, n = 101) and was pooled since no apparent differences were observed between the sampling sites. The species PCR positive samples were further categorized according to type of infection, gender and age group. The highest prevalence of malaria was found in males (85%, n = 96) and the prevalence of infections were highest in the age group of 21-40 years (66%, n = 75) followed by 0-20 years (18%, n = 20) (Table 1).

**Table 1:** Distribution of PCR positive samples from Jhapa and Banke districts in Nepal

Type of infection	Gender		Total		Age group	
			
	Male	Female		0-20 yrs	21-40 yrs	≥41 yrs
Pv	61(89%)	8(11%)	72(64%)	11(15%)	49(68%)	12(17%)
Pf	14(67%)	7(33%)	21(19%)	7(33%)	13(62%)	1(5%)
Pv+Pf	18(90%)	2(10%)	20(17%)	2(10%)	13(65%)	5(25%)

Total	96(85%)	17(15%)	113	20(18%)	75(66%)	18(16%)

Distribution of 113 positive samples based on species PCR according to infected species: Pv = *P. vivax*, Pf = *P. falciparum *and Pv+Pf = mixed infections, along with the gender and age group of the subjects.

### Prevalence of SNPs in *Pfcrt*

**Figure 2:**
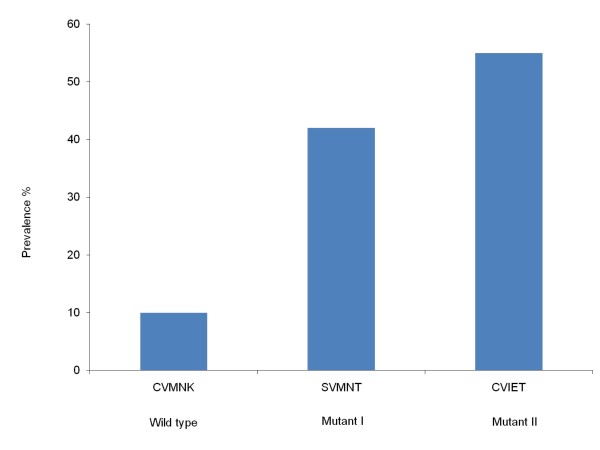
**Prevalence of haplotypes of *Pfcrt *in samples from Jhapa and Banke districts in Nepal**. Prevalence of haplotypes at codons 72-76 of the *Pfcrt *gene related to *P. falciparum *chloroquine resistance, including the mixed haplotypes (n = 31).

The prevalence of haplotypes at c72-76 could be determined for 31 out of 41 *P. falciparum *positive samples including mixed haplotypes (n = 2). The mutant haplotypes CVIET and SVMNT were identified in 55% (n = 17) and 42% (n = 13) of samples respectively while 10% samples (n = 3) contained the wild type CVMNK (Figure 2).

### Prevalence of SNPs in *Pfmdr1*

**Figure 3:**
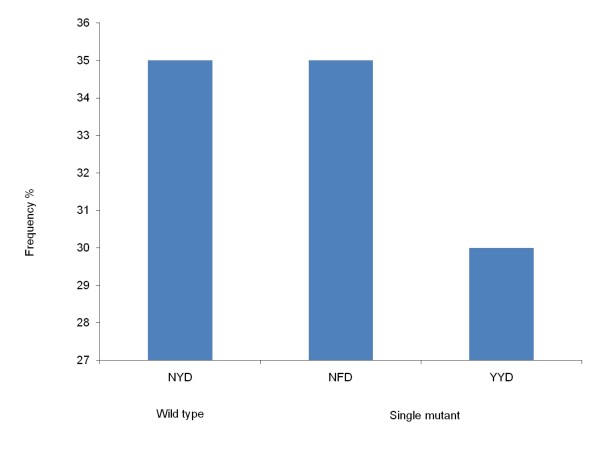
**Frequency of haplotypes of *Pfmdr1 *in samples from Jhapa and Banke districts in Nepal**. Frequency of constructed haplotypes of SNPs in the *Pfmdr1 *gene at c86, c184 and c1246 associated with *P. falciparum *chloroquine resistance (n = 26).

SNPs were examined at c86, c184 and c1246 in 33, 32 and 30 samples respectively. The prevalence of mutations in the *Pfmdr1 *gene were N86Y (33%, n = 11) and Y184F (44%, n = 14) including mixed infections (n = 1 for c86 and n = 3 for c184) while only wild type D1246 was observed (n = 30). Haplotypes could be constructed for three codons in 26 samples with different haplotypes identified: NYD (35%, n = 9), NFD (35%, n = 9) and YYD (30%, n = 8) (Figure 3).

### Prevalence of SNPs in *Pfdhfr and Pfdhps*

**Figure 4:**
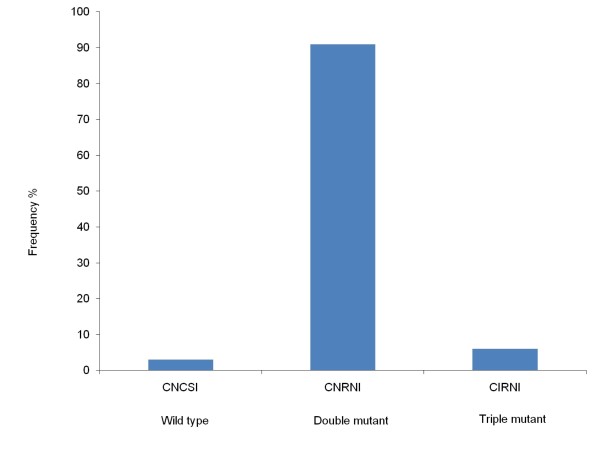
**Frequency of haplotypes of *Pfdhfr *in samples from Jhapa and Banke districts in Nepal**. Frequency of constructed haplotypes of SNPs in the *Pfdhfr *gene at c50, c51, c59, c108 and c164 associated with *P. falciparum *pyrimethamine resistance (n = 32).

For *Pfdhfr*, SNPs were examined at c50, c51, c59, c108 and c164 in 32 samples. Mutations were determined at N51I, C59R and S108N with the prevalence of 6% (n = 2), 97% (n = 31) and 97% (n = 31), respectively while only wild types were detected at c50 (n = 32) and c164 (n = 32). Haplotypes could be constructed for 32 samples and determined as either double mutants CNRNI (91%, n = 29), triple mutants CIRNI (6%, n = 2) and wild type CNCSI (3%, n = 1) (Figure 4).

**Figure 5:**
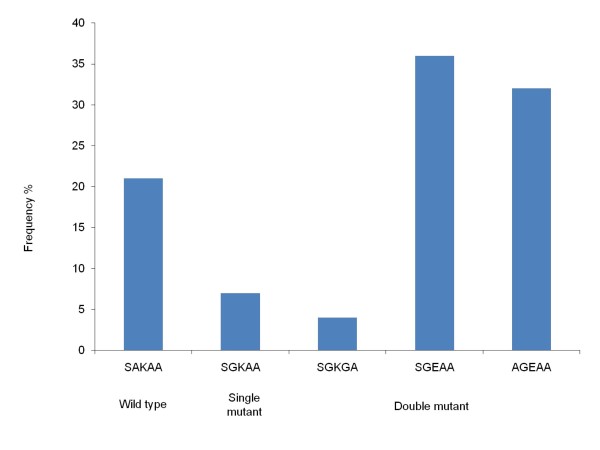
**Frequency of haplotypes of *Pfdhps *in samples from Jhapa and Banke districts in Nepal**. Frequency of constructed haplotypes of SNPs in the *Pfdhps *gene at c436, c437, c540, c581 and c613 linked with *P. falciparum *sulphadoxine resistance (n = 28).

SNPs at c436, c437, c540 were examined in 30 samples and at c581 and c613 in 31 samples of the *Pfdhps *gene, respectively. Majority of infections carried mutations at A437G (77%, n = 23), K540E (70%, n = 21) and S436A (33%, n = 10) while the A581G mutation was found in 3% of samples (n = 1) and wild type was observed only at c613 (n = 31). Haplotypes could be constructed for 28 samples, of which double mutants SGEAA (38%, n = 10) and AGEAA (33%, n = 9) were the major haplotypes, followed by wild type SAKAA (21%, n = 6), single mutant SGKAA (7%, n = 2) and double mutant SGKGA (4%, n = 1) (Figure 5).

When both *Pfdhfr *and *Pfdhps *haplotypes were jointly examined, haplotypes could be constructed for 27 samples. The main haplotypes were the quadruple mutant haplotypes CNRNI/SGEAA (33%, n = 9) and CNRNI/AGEAA (30%, n = 8), whereas the quintuple mutant haplotypes were determined low; CIRNI/AGEAA (4%, n = 1) and CIRNI/SGKAA (4%, n = 1).

### Prevalence of SNPs in *Pvmdr1*

**Figure 6:**
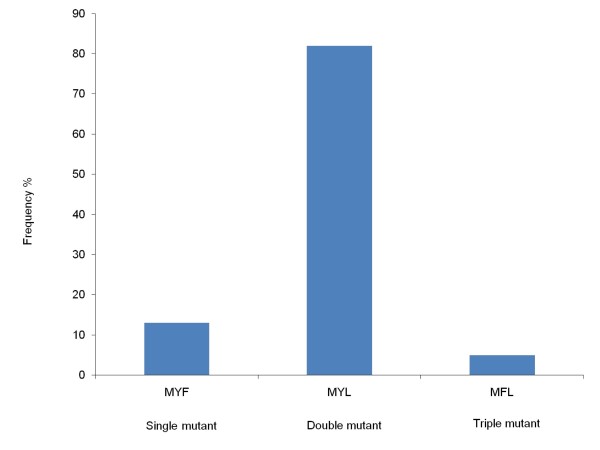
**Frequency of haplotypes of *Pvmdr1 *in samples from Jhapa and Banke districts in Nepal**. Frequency of constructed haplotypes of SNPs in the *Pvmdr1 *gene at c958, c976 and c1076 associated with *P. vivax *chloroquine resistance (n = 39).

Only a fraction of samples (n = 47) were sequenced by alignment of DNA sequence with the wild type Salvador 1 as reference strain and out of these, 39 samples had satisfactory results. Only a fragment of 784 bp covering the codons 958, 976 and 1076 of the *Pvmdr1 *gene had been sequenced. A novel mutation was identified at T958M in all samples while F1076L was observed in 37 samples (95%) and Y976F was only identified in two samples (5%). Including the T958M mutation in constructing haplotypes for c958, c976 and c1076, the most frequent haplotype was the double mutant haplotype MYL (82%, n = 32), whereas the single mutant MYF and the triple mutant MFL haplotypes were determined in 13% (n = 5) and 5% (n = 2) of the cases, respectively (Figure 6). Finally, novel SNPs were observed in two samples; one sample was found to contain a TTT to TCT mutation at c979 (F979S), and an ATG to GTG mutation at c980 (M980V). Further, one sample contained an AGT to AAT mutation at c1080 (S1080N).

### Prevalence of SNPs in *Pvdhfr *gene

**Figure 7:**
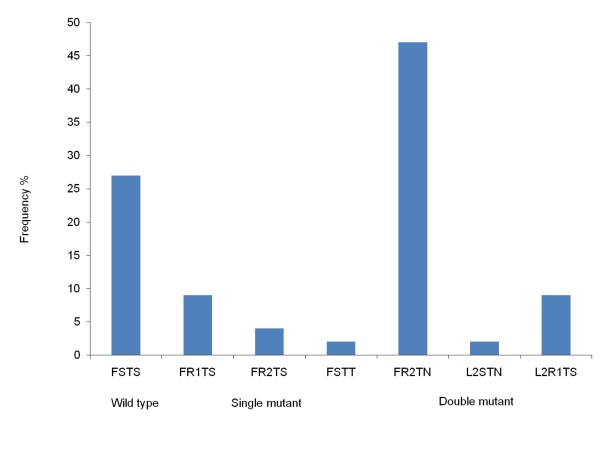
**Frequency of haplotypes of *Pvdhfr *in samples from Jhapa and Banke districts in Nepal**. Frequency of constructed haplotypes of SNPs in the *Pvdhfr *gene at c57, c58, c61 and c117 linked with *P. vivax *pyrimethamine resistance (n = 55).

SNPs were observed at c57, c58, c61 in 63 samples and at c117 in 65 samples respectively. *Pvdhfr *mutations were identified at S58R (68%, n = 43) followed by S117N/T (54%, n = 35) and F57L (11%, n = 7), whereas only wild type was determined at c61. Haplotypes could be constructed for 55 samples and the major haplotypes were the double mutant FR2TN (47%, n = 26) and wild type FSTS (27%, n = 15). Single mutant haplotypes FR1TS (9%, n = 5), FR2TS (4%, n = 2) & FSTT (2%, n = 1) and double mutant haplotypes L2R1TS (9%, n = 5) & L2STN (2%, n = 1) were also determined while triple and quadruple mutant haplotypes were not identified (Figure 7).

## Discussion

Several therapeutic efficacy studies were carried out in Nepal to determine the efficacy of currently used anti-malarial drugs. For *P. falciparum*, studies done from 1984 to 1990 had shown 38% *in vivo *resistance to CQ. Similarly, SP efficacy studies conducted between 1996 to 2000 showed 57-73% treatment failures in *P. falciparum *infections [36]. Contrarily, a trial performed in 2005 in Jhapa showed only 12% SP treatment failure [26]. In Nepal, changes in anti-malarial drug policies have been based on declining efficacy of first line drugs when the therapeutic efficacy fell below 90% [4]. In line with this, AL was introduced against *P. falciparum *in 2004 in 13 highly endemic districts. SP is still the first line of treatment in the other 52 endemic districts, however, ACT will gradually replace SP in the near future [4].

This study determined CQ and SP resistance at molecular level to explore whether the high level of *in vivo *CQ and SP resistance previously found in Nepal resulting in the recent policy change, is reflected at molecular level as well. Polymorphisms related to CQ resistance in *P. falciparum *were determined in both *Pfcrt *and *Pfmdr1*. Combined, the *Pfcrt *mutant haplotypes CVIET (55%) and SVMNT (42%) were determined in a total of 97% of samples indicating high levels of CQ resistance. These haplotypes were also the most common haplotype determined from the Indian state of Assam (33% CVIET and 62% SVMNT) in 2006 [37].

Studies done in Malawi [38] and Tanzania [39] had shown the re-emergence of CQ sensitive *P. falciparum *populations after the withdrawal of CQ as a first line drug. However, this was not observed here, most probably due to the current anti-malarial drug policy of Nepal which recommends CQ for laboratory unconfirmed *P. falciparum *infection [4]. Regarding *Pfmdr-1*, this study found a high prevalence of wild types N86 (67%), Y184 (56%) and D1246 (100%). Further, 35% of the constructed haplotypes were the haplotype NFD. This haplotype has previously been implied as a marker of emerging AL tolerance [35,40]. Therefore, it is important to continue the monitoring of this particular marker in Nepal now with AL as the first-line treatment in highly endemic areas.

Studies have indicated that Y976F is the most important marker of CQ resistance and F1076L of lesser importance [11,31,41]. The analysis of *Pvmdr1 *identified a novel mutation at T958M, which has only been reported in samples from Madagascar [42] and its role in CQ resistance is unknown. Likewise, mutation at Y976F was determined only in two samples (5%). This suggests the presence of limited prevalence of CQ resistant haplotypes in *P. vivax *population. Finally, SNPs at c979, c980 and c1080 were observed, which have not been described elsewhere and the implications of these are unknown.

Regarding SP resistance in *P. falciparum*, SNPs at c51, c59 and c108 of *Pfdhfr *were identified. The most frequent mutant haplotype was the double mutated CNRNI (91%), which was also the most frequent haplotype determined at 64% from Jhapa in 2005 [26]. Thus, it seems that the prevalence of double mutant haplotype has increased. Furthermore, in 2003/2004, the CNRNI haplotype was the most frequent haplotype determined at 50-91% and 74-80% from Utter Pradesh and Assam in India, respectively [43,44]. Likewise, the triple mutated CIRNI was detected at low prevalence (6%) in the current study and in concordance with the previous study done in Jhapa (prevalence of 10% and 3% in 2002 and 2005 respectively) [26]. Further, CIRNI was also determined low in samples from Utter Pradesh (5%) and Assam (10%) in 2003 [43,44], which indicates a low but consistent prevalence of the triple mutant haplotypes in the region. For *Pfdhps*, SGEAA (38%) and AGEAA (33%), the haplotypes that have been associated with treatment failure of SP [45] were the most prevalent in this study. In the study done in Assam in 2003/2004, SGEAA and AGEAA were determined in 2-6% and 11-17% of the samples, respectively [43,44]. This may suggest an increase of sulphadoxine resistant haplotypes in the region.

For *Pfdhfr *and *Pfdhps *combined, both quadruple (double mutations in *Pfdhps *and *Pfdhps*) and quintuple mutations (triple mutations in *Pfdhfr *and double mutations in *Pfdhps*) have been associated with treatment failures [43,46,47]. The present study determined quadruple mutations in 63% of samples. Further, the low prevalence (17%) of CNRNI-SGEAA/AGEAA in Assam in 2003 [43,44] may point towards the increase of prevalence of quadruple mutations in the region. Thus, this high prevalence may imply a high risk of SP treatment failure, which is in concordance with two previous studies done in Jhapa that recorded 12% and 21% treatment failures of SP [26,48].

Although SP is not used deliberately against *P. vivax *infections in Nepal, *P. vivax *is exposed to SP through mixed infections and/or incorrect diagnosis [20-22]. Regarding SNPs in the *Pvdhfr*, the double mutant FR2TN which is common in Uttar Pradesh (34%) and Assam (50%) [49], was the major mutant haplotype in the current study (47%) as well. The triple and quadruple *Pvdhfr *mutant haplotypes associated with high levels of *in vivo *SP resistance [50] were not found, indicating limited levels of *P. vivax *SP resistance in the study area.

The SNP patterns observed in *Pfcrt, Pfdhfr *and *Pvdhfr *genes in Nepal are similar to the studies performed in India, especially in the states that share borders with Nepal [37,43,49]. This indicates that either the parasites are under similar drug pressure in both countries or the free movement of people for work and other purposes between these countries are responsible for carrying parasites with similar drug resistance profiles. In this study, high percentages (85%) of the malaria positive were identified in males. Similar results were determined from Jhapa in 2005 (73%) and 2007 (75%) [26,51]. A study done in 2008 showed that around 37% of patients, particularly the males, had a history of a recent visit to India [52]. Although this study did not evaluate this aspect, it may be the possible cause for the higher percentage of infection in males and thus the similar patterns of SNPs in the resistance genes of *P. falciparum *and *P. vivax*.

A limitation to the present study is the small sample size. The majority of the samples collected on glass slides were found negative by PCR, which may be due to low parasitemia or misdiagnosis by microscopy or simply poor success in DNA extraction. Thus, the results should be interpreted with caution.

## Conclusions

This study has determined low molecular prevalence of resistance to CQ and SP in *P. vivax *and a high level of CQ and SP resistance in *P. falciparum *in a limited number of samples from two districts in Nepal. Therefore, CQ is most likely still effective in the treatment of *P. vivax *infections, but this remains to be tested *in vivo *while the change to ACT against *P. falciparum *infection seems justified at molecular level.

## Competing interests

The authors declare that they have no competing interests.

## Authors' contributions

SA carried out the sample collections, molecular analysis and drafted the manuscript. MLS and TTT participated in the design of the study, sequence alignment and data interpretation. MA^1^, CMOK and ICB funded the study, participated in the design and coordination and data interpretation. MA^2 ^coordinated the sample collection. All authors read and approved the final manuscript.
